# Long‐term results of definitive concurrent chemoradiotherapy using paclitaxel plus oxaliplatin in unresectable locally advanced esophageal cancer: a prospective phase II trial

**DOI:** 10.1002/cam4.897

**Published:** 2016-11-04

**Authors:** Tao Song, Xuebang Zhang, Min Fang, Ruping Zhao, Shixiu Wu

**Affiliations:** ^1^ Department of Radiation OncologyHangzhou Cancer HospitalHangzhouZhejiang310002China; ^2^ Department of Radiation OncologyThe First Affiliated Hospital of Wenzhou Medical UniversityWenzhouZhejiang325000China

**Keywords:** Chemoradiotherapy, esophageal cancer, oxaliplatin, paclitaxel, survival, toxicity

## Abstract

This prospective study aimed at assessing the efficiency and safety of concurrent chemoradiotherapy (CCRT) using paclitaxel (PTX) plus oxaliplatin (OHP) in unresectable locally advanced esophageal cancer patients. Between January 2006 and December 2010, 34 patients with unresectable locally advanced esophageal cancer were enrolled in this study. Radiotherapy was delivered with a daily fraction of 2.0 Gy to a total dose of 60 Gy over 6 weeks. Concurrent PTX (135 mg/m², *d*_1_) and OHP (130 mg/m², *d*_1_) were administered on Day 1 and Day 29 of radiotherapy. Of these patients, 76.5% completed the treatment course with a response rate of 73.5%, including eight (23.5%) patients with complete response and 17 (50.0%) patients with partial response. The median overall survival (OS) time was 23.7 months (range: 4.0–65.5 months) with 1‐, 3‐ and 5‐year OS rates were 64.3%, 36.6% and 25.8%, respectively. The median progression‐free survival (PFS) time was 21.2 months with 1‐, 3‐ and 5‐year PFS rates were 63.8%, 30.9% and 20.4%, respectively, During the CCRT course, the main grade 3 or greater acute toxicities were leukopenia (38.2%), esophagitis (14.7%), and dysphagia (11.8%), with late toxicity being infrequent. Although this study did not meet its primary endpoint, the application of CCRT with PTX and OHP in unresectable locally advanced esophageal carcinoma yielded satisfactory clinical outcomes and manageable toxicities.

## Introduction

Esophageal cancer is the third‐most common cause of cancer‐related death in China 1. Although esophagectomy is the optimal treatment option, a majority of patients are not suitable for resection at the time of presentation for locally advanced or metastatic disease and a large proportion of patients need receive radiotherapy 2. The Radiation Therapy Oncology Group (RTOG) 85–01 trial assessed the efficacy of definitive radiotherapy of 50.4 Gy plus concurrent chemotherapy with 5‐fluorouracil (5‐Fu) and cisplatin (CDDP) in patients with locally advanced esophageal cancer, which led to a long‐term survival rate of approximately 25%, which is similar to patients treated with surgery alone 3. This trial established definitive concurrent chemoradiotherapy (CCRT) as a standard treatment for locally advanced esophageal cancer patients who do not want surgery or are inappropriate for surgery as a result of technical or medical reasons. However, the incidence of treatment failure was still high in the CCRT arm of RTOG 85‐01. In addition, the acute toxicities associated with CDDP and 5‐Fu significantly increased and restricted the application of CCRT. It is necessary to explore more effective treatment regimens to improve the therapeutic ratio and reduce toxic reactions.

Paclitaxel (PTX), a broad‐spectrum cytotoxic drug, is widely used in the treatment of ovarian cancer, breast cancer, and lung cancer. Preclinical studies have shown that PTX could enhance radiation sensitivity of tumor cells, potentiate tumor response, and increase the therapeutic ratio of radiotherapy 4. In clinical studies substituting PTX for 5‐Fu, similar results were observed in terms of activity and efficacy, with a more favorable toxicity profile 5, 6, 7 and was proven to be tolerable for elderly esophageal cancer patients in our recent published report 8. Oxaliplatin (OHP) is a third‐generation platinum with a more favorable toxicity profile compared to CDDP. In vitro and in vivo studies have also shown OHP to be a potent radiosensitizing drug which could overcome resistance to CDDP 9. In 2007, we reported a phase II clinical trial which accessed the efficacy and toxicity of CCRT using PTX and platinum (15 patients received the treatment regime of PTX and OHP), our results showed that the combination of PTX/platinum and irradiation could improve the survival outcome for locally advanced esophageal cancer and the side effects were well tolerated 10.

Based on these backgrounds, we performed this prospective phase II clinical trial to evaluate the feasibility and efficacy of definitive CCRT with a modern double regimen composed of PTX and OHP in unresectable locally advanced esophageal cancer. The primary endpoint was objective tumor response rate (ORR) and the secondary endpoints were overall survival (OS), progression‐free survival (PFS), and toxic reactions.

## Patients and Methods

### Ethics statement

This study was an extension of our former phase II clinical trial which assessed the efficiency and safety of CCRT in the setting of PTX plus platinum mentioned above, we had only registered the presented clinical trial in the first affiliated hospital of Wenzhou Medical University in January 2006 and this study was approved by the Institutional Review Board (IRBs) of Wenzhou Medical University. Written informed consents were obtained from all the patients in accordance with the regulations of the IRBs.

### Eligibility

Patients were regarded eligible according to the following criteria: (1) histological diagnosis of esophageal cancer; (2) clinical stages II to IV disease according to the International Union Against Cancer (UICC, 2002) TNM stage criteria; (3) Eastern Cooperative Oncology Group (ECOG) PS of 0 or 1; (4) no evidence of severe organ dysfunction; (5) adequate bone marrow, renal, hepatic, cardiac, and respiratory function (hemoglobulin ≥9 g/dL, white blood cell ≥3000/*μ*L, neutrophil ≥1.5 × 10^9^/L, platelet counts ≥10 × 10^4^/*μ*L, serum creatinine < 1.5 mg/dL); and (6) no cancer treatments prior to enrollment.

**Figure 1:**
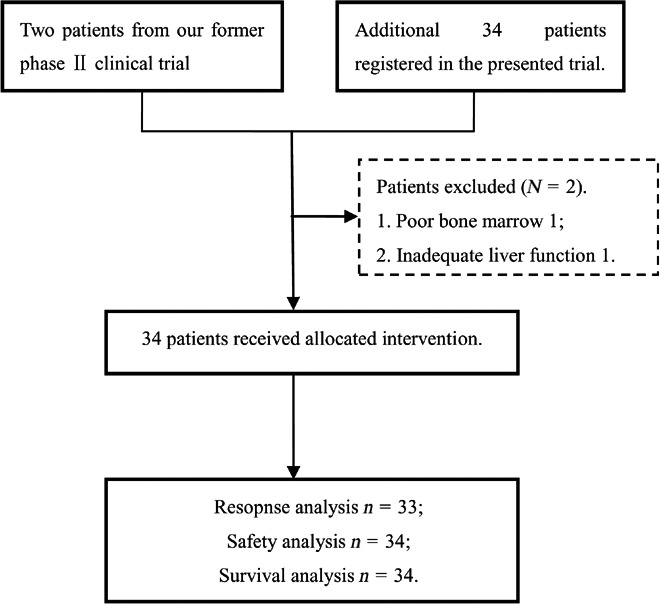
Consolidated standards of reporting trials diagram.

Patients were excluded if any of the following exclusion criteria were fulfilled: (1) prior treatments of chemotherapy or irradiation; (2) poor bone marrow, liver, and kidney functions, which would make chemotherapy intolerable; (3) contraindication for irradiation: complete obstruction of esophagus, deep esophageal ulcer, fistula to mediastinum, or hematemesis; (4) participating in other clinical trials; (5) pregnancy, breast feeding, or not adopting birth control; (6) clinically significant and uncontrolled major medical conditions including but not limited to: active uncontrolled infection, symptomatic congestive heart failure, unstable angina pectoris or cardiac arrhythmia, psychiatric illness/social situation that would limit compliance with study requirements (Fig. 1).

### Pretreatment evaluation

The pretreatment evaluation included history, physical examination, electrocardiography and assessment of bone marrow, renal and hepatic function. The extent of disease evaluation included endoscopy, barium esophagram, computed tomography (CT, required), magnetic resonance imaging (MRI), and positron emission tomography/CT (PET/CT, if available). Patients without an esophageal mass or enlarged lymph nodes documented by CT scan or MRI were required to undergo endoscopic ultrasonography (EUS) to demonstrate primary tumor invasion into the muscularis propria. Bronchoscopy was required for patients with lesions near the carina, to exclude tracheoesophageal fistula. Bone scans were performed if clinically indicated.

### Treatment schedule and dose modification

**Figure 2:**
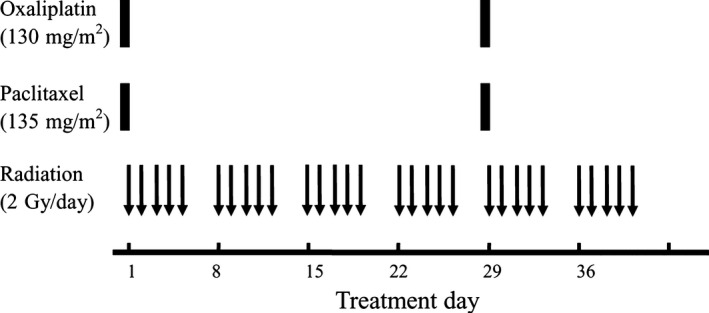
Treatment scheme.

PTX 135 mg/m^2^ was administered intravenously over 3 h on Day 1 and Day 29 with standard premedications. OHP (130 mg/m²) was given as a 2 h infusion at the same day with PTX. Radiotherapy started within 24 h of initiating chemotherapy using a high‐energy linear accelerator from Monday to Friday with weekends off (Fig. 2). The gross tumor volume (GTV) received 60 Gy (30 fractions at 2 Gy per fraction) and clinical target volume (CTV) was 40 Gy (20 fractions at 2 Gy per fraction). Radiotherapy was delivered in three‐dimensional conformal technique (3D‐CRT) and no intensity‐modulated radiotherapy was used. The definition of GTV, CTV, and dose–volume constraints of normal tissue in our institute was described previously 10, 11.

Chemotherapy was delayed for acute toxicities until recovery to grade ≤1, and/or the dose was reduced for grade 3 or higher hematological toxicity. PTX was reduced to 80% in the second course if any of the following occurred: grade 3 neutropenia with fever or grade 4 neutropenia. Granulocyte colony‐stimulating factor (G‐CSF) was used for the occurrence of febrile neutropenia. If the creatinine clearance decreased to <50 mL/min, the OHP dose was also reduced to 80%. Irradiation was interrupted for grade ≥3 esophagitis, grade 3 neutropenia with fever or grade 4 neutropenia. Radiotherapy was restarted when toxicities recovered to grade ≤2.

### Evaluation and follow‐Up

All of the patients were hospitalized and monitored weekly during the treatment course for acute toxicity. Physician‐reported hematological, esophageal, and pulmonary toxicities were evaluated according to the common toxicity criteria for adverse events version 3.0 (CTCAE v3.0). Clinical response was assessed according to the RECIST (Response Evaluation Criteria in Solid Tumors) system 4 weeks after the completion of treatment, and follow‐up was regularly carried out at 3‐monthly intervals in the first 2 years and at 6‐monthly intervals after 2 years. Late toxicity was evaluated based on the RTOG/European Organization for Research and Treatment of Cancer (EORTC) criteria 6 months after the completion of CCRT. Treatment failure was defined as any sign of recurrent disease, which could be local, distant, or both. We assessed failure models on post treatment CT (required), esophagogram, endoscopy, or PET/CT (if available) scans and compared those data with the original CT‐based radiation treatment plans.

### Statistical analysis

The Simon two‐stage mini‐max design was applied to determine sample size. It was calculated to achieve an expected response rate of 90% and a threshold response rate of 70% according to our previous study, with alpha (*α*) error of 0.05 and beta (*β*) error of 0.10, thus 32 eligible patients were needed. Considering some deviant cases, the planned accrual number was set to 34 patients 10, 12.

All of the statistical analyses were performed using SPSS version 16.0 software (SPSS Inc., Chicago, IL) with intention to treat analyses. OS was determined as the time (in months) between the first day of therapy and the last follow‐up or the date of death. PFS was calculated from the date of CCRT initiation to the date of documented failure (local recurrence or metastasis occurrence) or the date of the last follow‐up for those remaining. Survival curves were determined using the Kaplan–Meier method.

## Results

### Patients and tumor characteristics

Patients were recruited from January 2006 to December 2010; a total of 34 patients were assigned to receive CCRT with PTX and OHP (two patients treated with the same protocol were included in the analyses from our previous clinical trial). Patient and tumor characteristics are shown in Table 1. All patients had histologically confirmed squamous cell or adenocarcinoma of the esophagus. There were 32 men and 2 women, and the median age was 66 years. Patients’ performance status was evaluated as 0 in 14 patients and 1 in 20 patients. Four patients had clinical stage (cStage) II, 22 cStage III, and eight cStage IV esophageal cancers. Patient characteristics were discussed at a multidisciplinary meeting by a team of surgeons, gastroenterologists, radiation oncologists, medical oncologists, radiologists, and nuclear medicine physicians. Clinical decisions for CCRT were made due to metastatic diseases in eight patients, unresectable adjacent structure invasion (T_4_) in nine patients, and refusal of surgery in 17 including five patients with multiple comorbidities, eight patients with cervical esophageal cancer and four patients aged ≥70 years.

**Table 1:** Patients’ background characteristics

Characteristics	No. of patients (*N* = 34)	Percentage
Age (years)		
Median	66	
Range	39–80	
Gender		
Male	32	94.1
Female	2	5.9
ECOG performance status		
0	14	41.2
1	20	58.8
Weight loss in 6 months		
≤10%	19	55.9
>10%	15	44.1
T stage		
3	14	41.2
4	20	58.8
N stage		
0	14	41.2
1	20	58.8
M stage		
0	26	76.5
1	8	23.5
Clinical stage (AJCC 2002)		
II	4	11.8
III	22	64.7
IV	8	23.5
Histology on biopsy		
Squamous cell carcinoma	32	94.1
Adenocarcinoma	2	5.9
Histological differentiation		
Well differentiated	9	26.5
Fairly differentiated	14	41.2
Poorly differentiated	11	32.3
Tumor location		
Cervical+Upper thoracic	14	41.2
Middle thoracic	14	41.2
Lower thoracic+GEJ	5	14.7
Multisection	1	2.9
Length of tumor		
≤5 cm	17	50.0
>5 cm	17	50.0
CT scan	33	97.1
Barium swallow	30	88.2
Echoendoscopy	25	73.5
PET/CT scan	8	23.5

*N*, number of patients; ECOG, Eastern Cooperative Oncology Group; AJCC, American Joint Committee on Cancer; GEJ, gastroesophageal junction.

### Treatment compliance and toxicities

All patients completed the first cycle of chemotherapy with full‐dose intensity. In the second cycle of chemotherapy, 27 (79.4%) patients received the full‐dose regimens and 5 (14.7%) patients required a 20% dose reduction due to adverse events. The remaining two patients did not receive the second cycle of chemotherapy for developing lung metastasis during treatment (one patient) and getting fever after developing grade 4 leukopenia (another patient). Both patients also gave up radiation. Thirty‐one (91.2%) patients completed radiation, including five patients with radiation delay for adverse events. The remaining three patients gave up radiation for new distant metastasis (one patient, previously reported), treatment‐related toxicity (one patient, previously reported), and financial reason (one patient). Twenty‐six (76.5%) of 34 patients finished CCRT on schedule.

The acute toxicity profile of CCRT is listed in Table 2. Toxic reactions were assessed in all of the 34 patients. The most common hematologic toxicity was leukocytopenia, including eight (23.5%) patients with grade 3 and five (14.7%) patients with grade 4, respectively. Most patients recovered by using G‐CSF. Grade 3 thrombocytopenia was observed in six (17.6%) patients and three (8.8%) patients experienced grade 3 anemia. All patients got esophagitis including four (11.8%) patients with grade 3 and one patient with grade 4. Other grade 3 non‐hematologic toxicities included dysphagia (11.8), nausea/vomiting (8.8%), and fatigue (5.9%). No patients died of acute treatment‐related toxicities; no cardiac toxicities or hypersensitivity reactions related to PTX were reported. In terms of late toxicities, six (17.6%) patients exhibited all grade esophageal stenosis and five (14.7%) patients experienced radiation‐related pneumonitis. In general, the regimen of CCRT with PTX and OHP was well tolerated.

**Table 2:** Acute treatment‐related toxicities (*N* = 34)

	CTCAE Version 3.0				
Factor	Grade 1	Grade 2	Grade 3	Grade 4	≥Grade 3 (%)
Hematologic toxicity					
Leukocytopenia	6	11	8	5	38.2
Anemia	8	5	3	0	8.8
Thrombocytopenia	7	5	6	0	17.6
Non‐hematologic toxicity					
Esophagitis	6	23	4	1	14.7
Dysphagia	9	11	4	0	11.8
Mucositis	13	7	1	0	2.9
Diarrhea	8	5	2	0	5.9
Nausea/vomiting	18	8	3	0	8.8
Fatigue	5	6	2	0	5.9

CTCAE Version 3.0: Common Terminology Criteria for Adverse Events, Version 3.0.

### Tumor response to CCRT and failure pattern

Based on the study protocol, 14 out of 18 patients were observed as responders (CR+PR) in the first stage and we continued to the second stage for a total of 34 patients. However, one patient in the second stage could not be evaluated for response for the reason previously reported. Thus, a total of 33 patients were eligible for response evaluation based on the RECIST system, which was done 4 weeks after completion of treatment. Based on the intention‐to‐treat analysis, CR was observed in 8 (23.5%) patients, PR in 17 (50.0%) patients, SD in 6 (17.6%) patients, and PD in 2 (5.9%) patients. Objective tumor response rate (ORR) was 73.5% (34 patients). Moreover, 22 (64.7%) patients underwent biopsy to confirm the response. Of the 22 patients, tumor cells were not found in 13 patients, only heterocyst cells were found in five patients, and four patients had persistent disease according to the pathological reports. At the end of the last follow‐up, 27 (79.4%) patients experienced disease recurrence. Primary recurrent sites included: eight loco‐regional and local residual disease, 16 distant failure including 5 in liver, 3 in lung, 3 in bone, 1 in the adrenal gland, and 4 in non‐reginal lymph nodes. Three patients suffered treatment failure in both (loco‐regional and distant) sites.

### Survival outcomes

**Figure 3:**
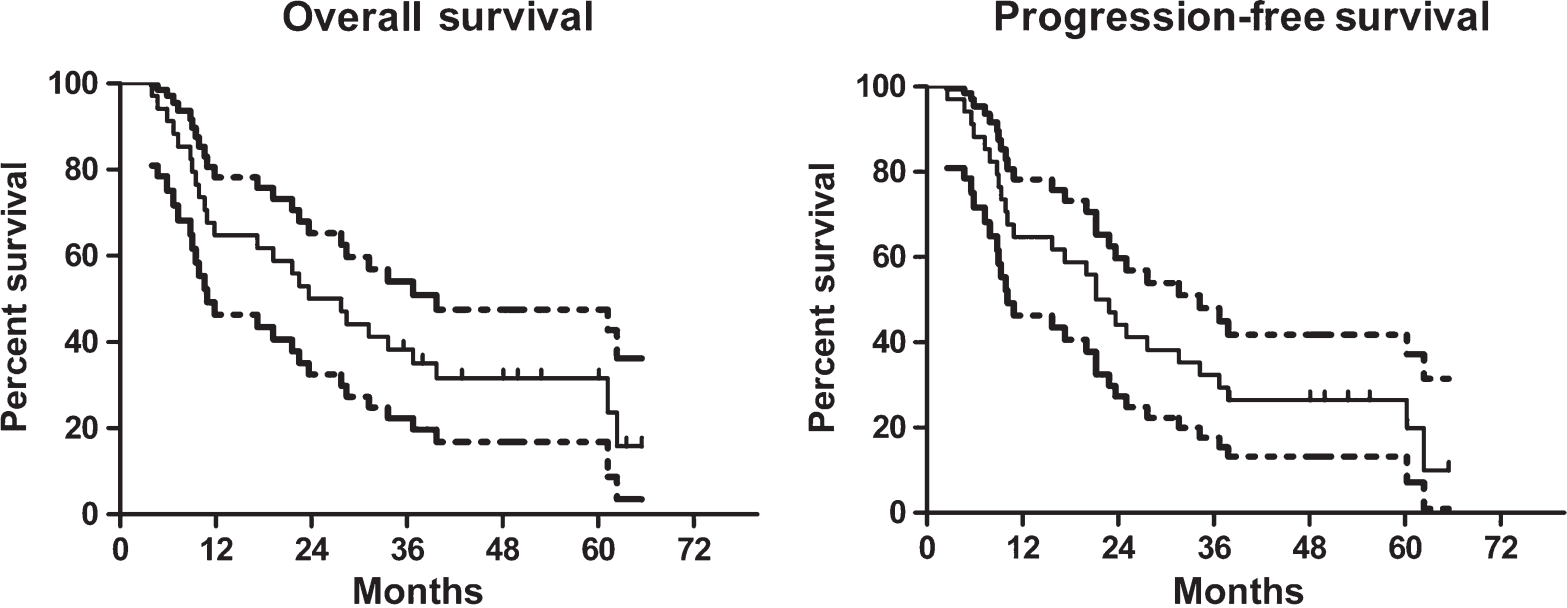
Overall survival and progression‐free survival with 95% confidence interval for patients treated with concurrent chemoradiotherapy using paclitaxel plus oxaliplatin.

The median follow‐up period was 49.8 months (range: 2.6–67.8 months). The median OS of the overall population was 23.7 months (95% CI: 13.9–33.5 months). The 1‐, 3‐ and 5‐year OS rates were 64.3% (95% CI: 0.486–0.808), 36.6% (95% CI: 0.219–0.545), and 25.8% (95% CI: 0.059–0.415), respectively. The median PFS time was 21.2 months (range: 2.6–65.6 months, 95% CI: 16.0–26.4 months), with 1‐, 3‐ and 5‐year PFS rates of 63.8% (95% CI: 0.486–0.808), 30.9% (95% CI: 0.141–0.447), and 20.4% (95% CI: 0.040–0.358), respectively (Fig. 3).

## Discussion

This study was conducted to assess the efficacy and safety of CCRT with PTX and OHP in patients with unresectable locally advanced esophageal cancer. Our results showed that the double regimen composed of PTX and OHP were effective and the treatment‐related toxicities were acceptable. Of the 34 patients enrolled in our study, 31 (91.2%) patients completed preplanned CCRT, including 27 (79.4%) patients who received full dose of chemotherapy. The full dose of chemotherapy completion rate in this study was much higher than in the RTOG 85‐01 trial, in which all cycles of chemotherapy could be administered as planned in 33 of 61 (54%) patients 3. The completion rate in our study was in line with other recent clinical trials. In the PRODIGE5/ACCORD17 trial, the treatment regime was delivered as planned in 124 (97%) of 128 patients in the 5‐Fu and CDDP groups and in 122 (93%) of 131 patients in the FOLFOX group. Full completion of all chemotherapy cycles was achieved in 93 (71%) of 131 patients in the FOLFOX group and 97 (76%) of 128 patients in the 5‐Fu and CDDP groups 13. In a phase II clinical trial which investigated the efficacy of CCRT in the setting of PTX and CDDP, Tang et al. reported that 89.5% (68/76) and 63.2% (48/76) patients completed ≥2 cycles and all four cycles of chemotherapy, respectively 14.

Clinical response evaluation showed that the ORR in this study was 73.5%. Although the response rate did not achieve the expected rate of 90%, our results were comparable to those reported trials using CCRT with other regimens. Kodaira et al. reported that the ORR rate in patients treated with 5‐Fu and nedaplatin was 76%, including CR in 12 and PR in 7 patients 15. Thierry et al. reported ORR rates of 66% and 65% in patients treated with FOLFOX and 5‐Fu/CDDP, respectively 13. In this study, most of the patient characteristics were recorded based on CT scans and barium swallows, making it difficult to discriminate residual thickening of the esophageal wall caused by tissue reactions after CCRT (inflammation, necrosis, and fibrosis) or residual tumor, so we might underestimate the clinical response rate. Moreover, of the 22 patients who underwent biopsy in our study, tumor cells were not found in 13 patients, only heterocyst cells were detected in five patients, and four patients had persistent disease after completion of CCRT, thus a total of 18 (81.8%) patients would be considered as respond to the treatment.

The most frequent Grade 3–4 acute toxicities in the current regimen were leukopenia and esophagitis. The incidence of leukopenia and esophagitis of grade 3–4 was 38.2% (13/34) and 14.7% (5/34), respectively. No patient died of acute treatment‐related toxicities. The profile of acute toxicity during CCRT was consistent with that reported in our previous phase II trial in the setting of PTX/platinum and irradiation 10. In another phase I/II study which assessed the safety of neoadjuvant chemoradiotherapy with docetaxel and OHP in patients with locally advanced adenocarcinoma of the esophagogastric junction, results also indicated that the treatment regimen based on docetaxel and OHP was effective and showed a good toxicity profile 16. Compared with CDDP‐based chemotherapy plus 60 Gy radiotherapy, our recent systemic review and pooled analysis which enrolled 1915 patients from 26 clinical studies also showed that the most common acute toxicities of grade 3 or higher for CDDP‐based CCRT were hematologic toxicities, the most severe grade 3 or higher radiation‐related acute toxicity was esophagitis, and the pooled incidence of esophagitis was 12.8% which was in line with this cohort 17. In terms of late toxicities, we noted two (5.9%) patients experienced grade 3 esophageal stenosis and five patients got grade 1–2 pneumonitis. The late toxicities were also lower than that observed in RTOG 85‐01, in which 21.3% patients in the CCRT arm experienced late esophageal morbidity 3. In general, our treatment regimen was well tolerated.

The median OS in this study was 23.7 months, which was better when compared with the 14.1 month median OS in the CCRT group of the RTOG 85‐01 trial 3 and the 13 month median OS in high‐dose group of the RTOG 94‐05 trial 18 . The survival outcomes in our study were also comparable to latest results from other clinical trials in the CCRT setting with modern chemotherapy regimens. Li et al. reported an estimated median OS time of 22.6 months and an OS rate at 3 years of 36.7% in patients receiving CCRT with docetaxel and CDDP 19. Ewout et al. reported that the median OS in the group of patients who received chemoradiotherapy as definitive treatment was 15 months, with 1‐ and 3‐year OS rates of 63% and 24%, respectively 20. In the PRODIGE5/ACCORD17 trial, Thierry et al. reported that the median OS was 20.2 months in the FOLFOX group and 17.5 months in the 5‐Fu and CDDP groups. OS rates at 3 years was 19.9% in the FOLFOX group and 26.9% in the 5‐Fu/CDDP group, which is inferior to the 36.6% observed in our study 13.

This study has several limitations. Firstly, most of our patients had squamous cell carcinoma. As squamous cell carcinoma and adenocarcinoma of the esophagus differ in terms of epidemiology, location, and pathways of progression, it is difficult to extrapolate our results for patients with adenocarcinoma. Secondly, due to the socioeconomic concerns in mainland China, PET/CT was not routinely used in this study. The clinical stage and clinical response evaluation were mainly done by CT scans. The results were not as accurate as that obtained by PET/CT. Additionally, patients in this cohort were recruited over a long period of time, and some unmeasurable factors might have effects on the final results, although there was strict accordance with the treatment protocol.

In conclusion, CCRT with PTX and OHP can be safely delivered in unresectable locally advanced esophageal cancer. The toxicities associated with therapy were tolerated and manageable. It has the potential to improve survival outcomes in esophageal carcinoma and should be validated in future large‐sample clinical studies.

## Conflict of Interest

The authors indicated no potential conflicts of interest for this study.
